# Quantifying changes in soil organic carbon density from 1982 to 2020 in Chinese grasslands using a random forest model

**DOI:** 10.3389/fpls.2023.1076902

**Published:** 2023-05-08

**Authors:** Jie Chen, Asim Biswas, Haohai Su, Jianjun Cao, Shuyan Hong, Hairu Wang, Xiaogang Dong

**Affiliations:** ^1^ College of Geography and Environmental Science, Northwest Normal University, Lanzhou, China; ^2^ School of Environmental Sciences, University of Guelph, Guelph, ON, Canada; ^3^ Key Laboratory of Eco-functional Polymer Materials of the Ministry of Education, Northwest Normal University, Lanzhou, China

**Keywords:** soil organic carbon, emission peak, carbon neutrality, carbon pool, climate change

## Abstract

China has the second-largest grassland area in the world. Soil organic carbon storage (SOCS) in grasslands plays a critical role in maintaining carbon balance and mitigating climate change, both nationally and globally. Soil organic carbon density (SOCD) is an important indicator of SOCS. Exploring the spatiotemporal dynamics of SOCD enables policymakers to develop strategies to reduce carbon emissions, thus meeting the goals of “emission peak” in 2030 and “carbon neutrality” in 2060 proposed by the Chinese government. The objective of this study was to quantify the dynamics of SOCD (0–100 cm) in Chinese grasslands from 1982 to 2020 and identify the dominant drivers of SOCD change using a random forest model. The results showed that the mean SOCD in Chinese grasslands was 7.791 kg C m^−2^ in 1982 and 8.525 kg C m^−2^ in 2020, with a net increase of 0.734 kg C m^−2^ across China. The areas with increased SOCD were mainly distributed in the southern (0.411 kg C m^−2^), northwestern (1.439 kg C m^−2^), and Qinghai–Tibetan (0.915 kg C m^−2^) regions, while those with decreased SOCD were mainly found in the northern (0.172 kg C m^−2^) region. Temperature, normalized difference vegetation index, elevation, and wind speed were the dominant factors driving grassland SOCD change, explaining 73.23% of total variation in SOCD. During the study period, grassland SOCS increased in the northwestern region but decreased in the other three regions. Overall, SOCS of Chinese grasslands in 2020 was 22.623 Pg, with a net decrease of 1.158 Pg since 1982. Over the past few decades, the reduction in SOCS caused by grassland degradation may have contributed to soil organic carbon loss and created a negative impact on climate. The results highlight the urgency of strengthening soil carbon management in these grasslands and improving SOCS towards a positive climate impact.

## 1 Introduction

The soil organic carbon (SOC) pool is the largest organic carbon pool in terrestrial ecosystems and the backbone of soil health, contributing greatly to soil fertility and quality and the functionality of various ecosystems (Lal, 2004; Zhao et al., 2018). Even a small reduction in SOC can lead to large amounts of CO_2_ emissions, and exacerbate global warming (Fan et al., 2008; Wang et al., 2011). Soil organic carbon density (SOCD) is an important index to measure soil organic carbon storage (SOCS) (Wu et al., 2019). Information on the spatiotemporal variability of SOCD is critical for understanding the global carbon budget and adjusting land-use management policies, around CO_2_ emission reduction and global warming mitigation (Wu et al., 2019). Grasslands store approximately 34% of the total soil carbon and 10% of SOC in global terrestrial ecosystems (Scurlock et al., 2002; Corona et al., 2016; Yuan et al., 2021). With a mean SOCD of 7.6–14.1 kg C m^−2^ and SOCS of 278.9–591.5 Pg, global grasslands have strong carbon sequestration potential and play a critical role in regulating the global carbon cycle and climate (King et al., 1997; Prentice et al., 2001; Piao et al., 2009; Wei and Fang, 2009; Yang et al., 2021).

China has the second-largest grassland area in the world, accounting for 6%–8% of the global grassland area and 41% (3.95 × 10^6^ km²) of China’s total territory (Fan et al., 2008; Deng et al., 2017; Yang et al., 2021). Understanding the dynamics of SOCD in Chinese grasslands not only enables the assessment of the carbon budget at a national scale but also helps to evaluate the contribution of Chinese grasslands to global grasslands’ soil carbon balance (Zhang et al., 2016). Process-based biochemical models and data-driven empirical models are two common methods for simulating and assessing SOCD dynamics. Biochemical models, such as the Rothamsted Carbon (RothC) model, the CENTURY model, and the denitrification–decomposition (DNDC) model are typically used for SOCD dynamic assessment (Stockmann et al., 2013; Lee and Viscarra Rossel, 2020), but differences in model mechanisms and input parameters could lead to diverse results (Li H et al., 2022). Empirical models are built on the soil-forming factors equation, which generally requires sufficient field survey data to establish relationships between SOCD and climatic, biological, soil, topographic, and other soil-forming factors (McBratney et al., 2003; Zhang Y et al., 2022). In practice, the accuracy of this method depends on the amounts of field survey data, while the availability of sufficient field survey data remains limited by various conditions (Yang et al., 2016). In recent years, machine learning algorithms (e.g., classification and regression tree, artificial neural network, and random forest) have been used extensively to build empirical models and simulate SOCD (Yang et al., 2016; Wadoux et al., 2020). Many researchers have employed the space-for-time substitution processes to simulate SOCD dynamics by constructing empirical models based on machine learning algorithms (Szatmári et al., 2019; Zhang W et al., 2022). Based on a certain number of observed samples, the spatiotemporal dynamics of SOCD can be simulated using time-varying covariates, which can reduce challenges in obtaining measurement data historically. The random forest (RF) model can effectively handle non-linear relationships, can reduce overfitting, and has shown good predictive power in many studies (Breiman, 2001; Singh et al., 2017; Keskin et al., 2019; Zhu et al., 2023). Therefore, it has been often used for modeling and mapping SOCD (Yang et al., 2016; Li H et al., 2022).

Climate, elevation, soil texture, and human activities are key factors driving SOCD change and are often used as input into models to assess and predict SOCD dynamics (Hartley et al., 2021; Zhang Y et al., 2022; Liang et al., 2019; Li H et al., 2022). For example, the increased temperature can accelerate the SOC decomposition rate and decrease SOCD (Ofiti et al., 2021). Furthermore, it can promote microbial activity and accelerate the accumulation of microbial assimilative synthesis products, promoting the formation of stable soil carbon (Hao et al., 2021). Changes in precipitation can control litter input and soil respiration by influencing soil water content and soil microbial activity, which further affects the accumulation of SOC (Zhang et al., 2016). It is reported that wind speed, sunshine duration, and humidity also affect SOCD (Lu et al., 2013; Lei et al., 2019; Huang et al., 2022). As an important anthropogenic factor, grazing and its intensity (standard sheep unit ha^−1^ year^−1^) can disturb the carbon balance in ecosystems by affecting SOCD (Zhang et al., 2016; Zhou G et al., 2017; Li B et al., 2022). For example, slight and heavy grazing intensity may result in SOC loss, while moderate grazing intensity probably promoted SOC accumulation (Jiang et al., 2020; Zhang M et al., 2018; Xie and Wu, 2016). The net primary production (NPP) of plants and normalized difference vegetation index (NDVI) can drive SOCD change. NPP is a major determinant of terrestrial carbon sinks and an important regulator of the ecosystem carbon cycle (Zhang et al., 2016). NDVI reflects vegetation growth and biomass, and the influence of vegetation on SOCD changes (Zhang C et al., 2021; Liu et al., 2019). Overall, SOCD dynamics are regulated by the long-term equilibrium state of biophysical and chemical processes (Plante et al., 2014). These processes are closely linked to and interact with climate change, vegetation growth, environmental change, and human activities, which make SOCD vulnerable to external factors (Jobbágy and Jackson, 2000; Gaitan et al., 2019; Li H et al., 2022).

The overall objective of this study was to explore the spatiotemporal dynamics of SOCD over the past four decades in Chinese grasslands and to identify the dominant factors driving SOCD change. Specifically, a total of 15 factors, comprising mean annual temperature (MAT), mean annual precipitation (MAP), NPP, NDVI, elevation, aspect, sand, silt, clay, mean annual wind speed (MAWS), evapotranspiration (ET), sunshine hours (SH), relative humidity (RH), large livestock population (LLP), and sheep population (SP), were used to establish the relationship with SOCD using an RF model. The relationship was then used to quantify the spatiotemporal variation of SOCD in Chinese grasslands from 1982 to 2020. Dominant factors driving SOCD change were then identified based on their relative importance. We hypothesized that (i) the SOCD of Chinese grasslands showed a net increasing trend during 1982–2020, and (ii) the SOCS of Chinese grasslands gradually increased over time as a response to the conservation programs implemented over the past decades to control grassland degradation (Lu et al., 2018). The information can provide the basis for land-use adjustments and ecological projects and facilitate the realization of China’s ambitious national emissions reduction targets.

## 2 Materials and methods

### 2.1 Grasslands in China

**Figure 1:**
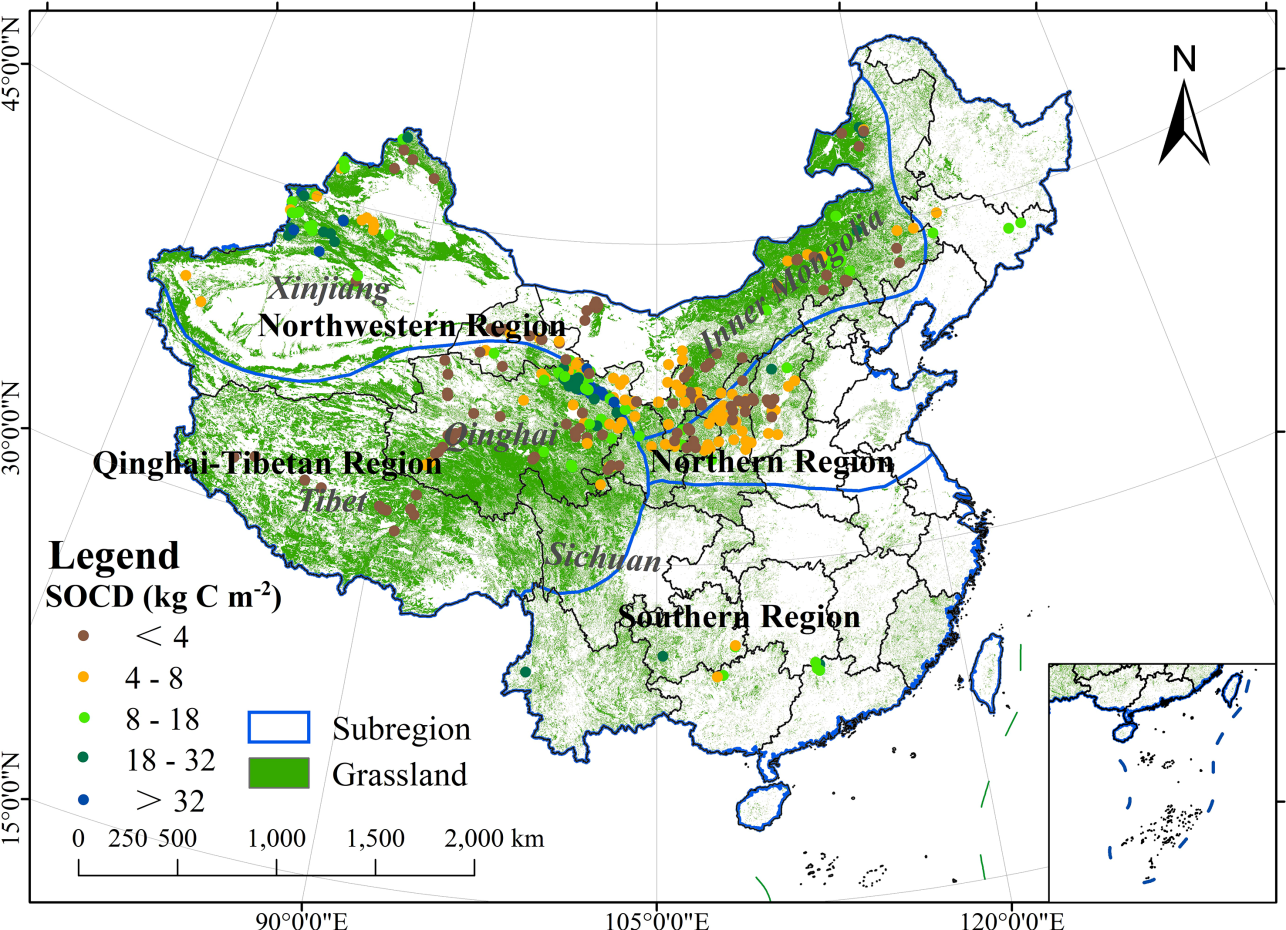
Spatial distribution of soil samples across Chinese grasslands.

According to the bulletin of the third national land survey, in 2020, Chinese grasslands covered an area of 2.645 × 10^6^ km^2^, of which 80.59% were natural pastures and 0.22% were cultivated grasslands. Grasslands in China are mainly distributed in six provinces (Tibetan Autonomous Region, Inner Mongolia Autonomous Region, Xinjiang Uygur Autonomous Region, Qinghai Province, Gansu Province, and Sichuan Province), accounting for 94% of the total grassland area. China is a vast country with complex and diverse climate types, and SOCD is unevenly distributed (Zhang Y et al., 2022). To better investigate the distribution and variation of SOCD, based on climate conditions, Chinese grasslands were divided into four regions in this study: northwestern, northern, southern, and Qinghai–Tibetan regions (**Figure 1**). The northern and southern regions are in the north and south of the monsoon climate zone, respectively. The northwestern region is in the non-monsoon climate zone, and the Qinghai–Tibetan region is a unique geographical area with a distinctive climate type (Zhang Y et al., 2022).

### 2.2 Data and processing

#### 2.2.1 Soil measurement sample data

We obtained a total of 552 measured grassland SOCD data (0–100 cm) from “A dataset of carbon density in Chinese terrestrial ecosystems (2010s)” for RF modeling (Xu et al., 2019). Each data point contained ecosystem type, location (longitude and latitude), the value of SOCD, sampling time, data type, and data source. There are two types of soil data: (a) Direct data, referring to SOCD data obtained directly from experimental tests, and SOCD and other relevant parameters (soil bulk density, SOC content, and soil depth) from literature. Among these, for soil depth< 100 cm, the SOCD of 0–100 cm was calculated according to the actual depth, and for those samples with depth > 100 cm, only 0–100 cm was selected. (b) Indirect data, which means there are no SOCD data in the experiments or literature, and the relevant parameters are incomplete, thus needing some deductions.

SOCD information from indirect data was derived according to the following rules (Xu et al., 2019): (a) Soil samples lacking bulk density were calculated using a pedotransfer function. (b) For soil samples without SOC, but with soil organic matter content, a conversion factor of 0.58 was used to convert soil organic matter to SOC. (c) For soil samples without gravel content, the mean value of known soil types was used instead. Detailed collection and calculation processes on SOCD can be found in Xu et al. (2019).

The dataset was subjected to strict quality control in the process of literature selection, data extraction, and collation to make the data reliable. We selected 552 sample values for the sampling period between 2009 and 2014 representing the soil data year 2010 for subsequent RF model construction. In addition, we selected 100 soil samples collected between 2000 and 2005 to validate the temporal accuracy of simulated SOCD data. The spatial distribution of soil samples used for modeling is shown in **Figure 1**.

#### 2.2.2 Land-use datasets

To identify the area and distribution of Chinese grasslands in various years, we collected land-use data for 1980, 1990, 2000, 2010, and 2018 from the Resource and Environment Science and Data Center (https://www.resdc.cn/). The original spatial resolution of the land-use data was 1:100,000, and we resampled them to 30 × 30 m in raster formats for further analysis. To match the time of our study, we treated the land-use data of 1980 as the land-use data in 1982, and the land-use data of 2018 as the land-use data in 2020. The land-use data were divided into six primary types, namely, woodland, farmland, grassland, water body, built-up land, and unused land. It should be noted that gobi, sandy land, swampland, gravel and rock, bare land, and other unused land in the original land-use data were uniformly classified as unused land.

#### 2.2.3 Data for SOCD modeling

We used a total of 15 factors (MAT, MAP, NPP, NDVI, MAWS, ET, SH, RH, elevation, aspect, sand, silt, clay, LLP, and SP) to model SOCD in this study. Details of the data source for all modeling factors are presented in **Table 1**. These factors represented climate, topography, soil texture, vegetation, and anthropogenic factors. Of these, we considered topography factors (elevation and aspect) and soil texture factors (sand, silt, and clay) as static factors and the rest as dynamic factors. We resampled all the data at 1-km spatial resolution using the ArcGIS 10.2 software in the Albers_WGS_1984 coordinate system.

**Table 1:** Data sources for the modeling factors.

Variable	Data	Source	Original resolution	Final resolution
NDVI	Growing-season NDVI over China during 1982–2020	Wang (2021)	0.05°	1 km
NPP	Global annual GPP/NPP dataset (1982–2018)	Yuan et al. (2010) Zheng et al. (2019)	0.05°	1 km
	MODIS products (2019–2020)	(https://www.nasa.gov/)	1 km	1 km
MAT, MAP	Monthly temperature and precipitation dataset for China (1982-2020)	Peng et al. (2019)	1 km	1 km
MAWS, RH, SH	Annual data of basic meteorological stations (1982–2015) Daily dataset of Chinese terrestrial climate data V3.0 (2016–2020)	Sun et al. (2014); Sun et al. (2015); Sun et al. (2016)	1 km	1 km
		(http://data.cma.cn/wa)	1 km	1 km
ET	The transpiration-to-evapotranspiration ratio data (1982–2015)	Niu et al. (2020)	1 km	1 km
	Daily dataset of Chinese terrestrial climate data V3.0 (2016–2020)	(http://data.cma.cn/wa)	1 km	1 km
LLP, SP	National Bureau of Statistics of China (1982–2020)	(http://www.stats.gov.cn/)	Vector	1 km
Elevation	Shuttle Radar Topography Mission (DEM)	(https://www.resdc.cn/)	1 km	1 km
Aspect	Shuttle Radar Topography Mission (DEM)	Calculated from elevation	1 km	1 km
Sand, silt, and clay	Data of soil texture in China	(https://www.resdc.cn/)	1 km	1 km

NDVI, normalized difference vegetation index; NPP, net primary production; MAT, mean annual temperature; MAP, mean annual precipitation; MAWS, mean annual wind speed; SH, sunshine hours, RH, relative humidity; LLP, large livestock population; SP, sheep population.

The MAT and MAP data from 1982 to 2020 were derived from a 1-km monthly temperature and precipitation dataset for China. We used MATLAB to synthesize the month-by-month data into annual data by calculating the mean value of 12 months for each raster in a year. SH, RH, and MAWS data for 1982–2015 were obtained from the 1-km resolution annual relative humidity, sunshine hours, and mean wind speed datasets for China, while for 2016–2020, they were generated based on meteorological station data and then interpolated in ArcGIS 10.2 using ordinary kriging. The ET data for 1982–2015 were from the spatial–temporal continuous dataset of the transpiration-to-evapotranspiration ratio in China. Based on the meteorological station data, we calculated ET for 2016–2020 using the Penman–Monteith formula and then interpolated it to the study area.

The vegetation factors include NDVI and NPP. The NDVI data ranged from 1982 to 2020 with a raw spatial resolution of 0.05° and a temporal resolution of 1 day. It was prepared by averaging the data for the first 15 days and the last 15 days of each month and then using the maximum value synthesis method to generate monthly average NDVI data for China. We collected NPP data for 1982–2018 from the global NPP/GPP dataset and NPP data for 2019–2020 from the MODIS product dataset.

LLP and SP are anthropogenic factors. Based on the data recorded in the Statistical Yearbook from 1982 to 2020, we converted the LLP and SP data to a raster format using the “Polygon to Raster” tool and resampled it to a spatial resolution of 1 km using ArcGIS 10.2 software.

Topography data included elevation and aspect data, and we obtained them from the Shuttle Radar Topography Mission (DEM). The aspect data were calculated from DEM data in ArcGIS 10.2. Soil texture was obtained from Resource and Environment Science and Data Center and consisted of sand, silt, and clay.

### 2.3 Methods

#### 2.3.1 Random forest model

RF model, one of the most popular methods for simulating SOCD spatiotemporal dynamics, has a consistent predictive power even in complex situations (Yang et al., 2016; Wadoux et al., 2020). It is created by using bootstrap samples of training data and random feature selection of the tree and has been effectively used for numerical prediction. It can also handle multivariate interactions and non-linear relationships as well as can estimate uncertainties in sparse samples and marginal regions, thus improving the prediction accuracy (Sreenivas et al., 2014; Vaysse and Lagacherie, 2017; Gyamerah et al., 2020). Due to the limitations of obtaining historical data on soil samples, we used time-varying factors as predictors to simulate SOCD in various years. This is a method of the space-for-time substitution being commonly used in a previous study concerning long-term SOC dynamics modeling (Padarian et al., 2022).

In the RF model running, we tested the model by setting three parameters (the maximum depth of trees, the random state, and the number of estimators). To further assess the accuracy of the model predictions, we used 10-fold cross-validation procedures to calculate the coefficient of determination (*R* ^2^) and root mean square error (RMSE) (Yang et al., 2020). The detailed description of the 10-fold cross-validation process and the calculation equations of *R* ^2^ and RMSE can be found in Zhu et al. (2019).

#### 2.3.2 Mann–Kendall test

The Mann–Kendall (MK) trend test is suitable for testing linear trends and nonlinear trends (i.e., the rate of change in each period has obvious change, as well as a certain regularity) (Shadmani et al., 2012). The *Z*-value and *p*-value are important parameters in measuring the trend. *Z* > 0 is an upward trend and the opposite is a downward trend (Yang et al., 2017). The *p*-value represents the significance of the trend (Shadmani et al., 2012; Yang et al., 2022). It is calculated as follows (Tošić, 2004):


(1) $$Z=\{\begin{array}{c} \frac{S-1}{\sqrt{Var\left(S\right)}},\;\;S>0\\ \;\;\;\;0,\;S=0\\ \frac{S+1}{\sqrt{Var\left(S\right)}},\;\;S<0 \end{array}$$

where *S* is the test statistic and *Var(S)* is the variance of the statistic ***S***, which can be described as:


(2) $$S=\sum_{i=1}^{n-1}\sum_{i=i+1}^{n}sgn\left(x_{j}-x_{i}\right)$$

where *x_j_* and *x_i_* are the time series values for years ***j*** and ***i***, respectively; *sgn* is the sign function.


(3) $$sgn(x_{j}-x_{i})=\{\begin{array}{c} 1,x_{j}-x_{i}>0\\ 0,x_{j}-x_{i}=0\\ -1,x_{j}-x_{i}<0 \end{array}$$

#### 2.3.3 Calculation of soil organic carbon storage

SOCS can be calculated using the following formula (He et al., 2021):


(4) SOCS=SOCD×Area

where *SOCD* is the mean soil organic carbon density of grasslands in each geographic region; *Area* is the area of grassland in each geographic region.

### 2.4 Modeling and testing

**Figure 2:**
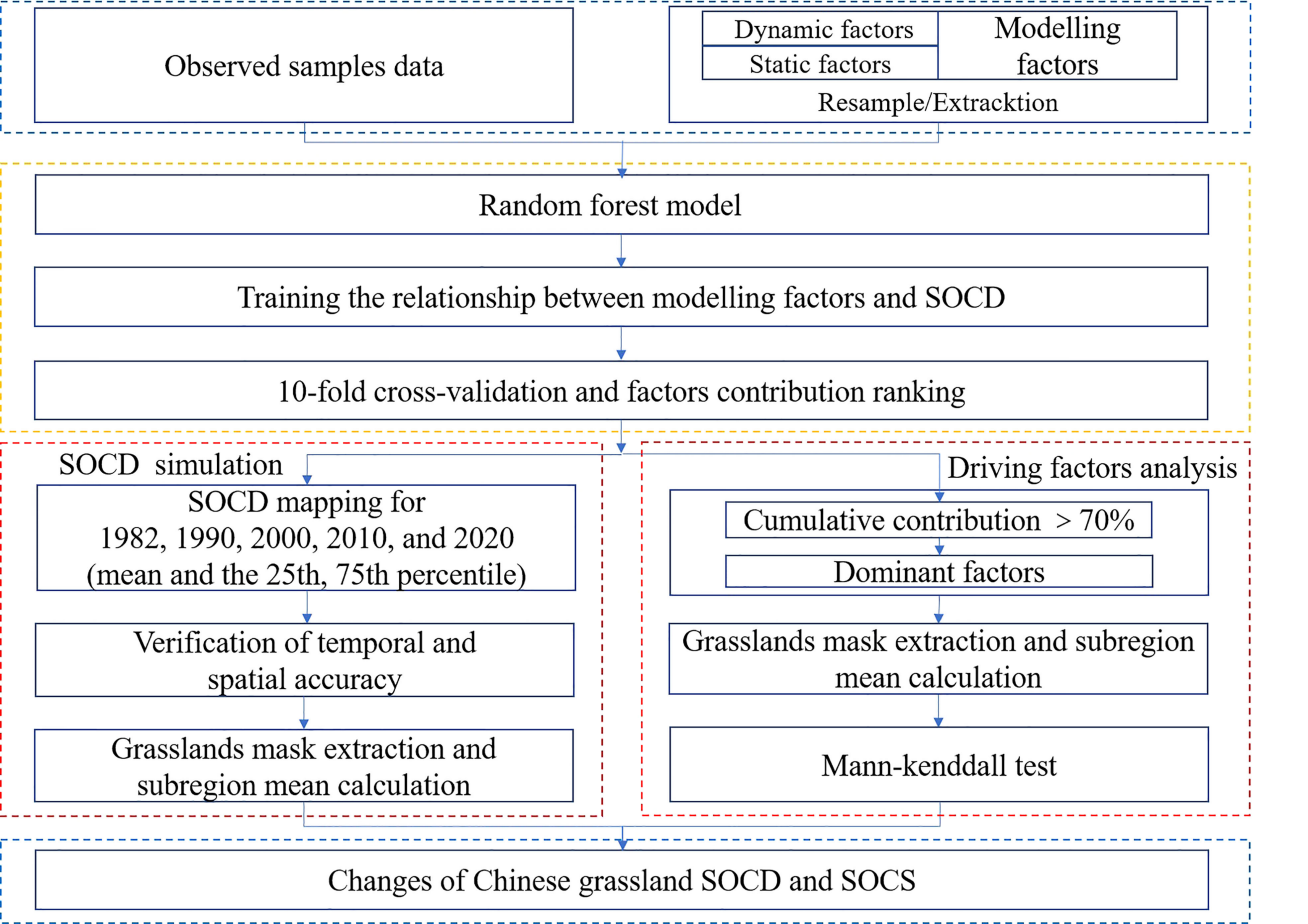
Framework diagram of this study. SOCD, soil organic carbon density; SOCS, soil organic carbon storage.

The basic flow of SOCD simulation modeling and subsequent analysis are presented in **Figure 2**. Based on 552 observed data and the modeling factors, we built a grassland SOCD prediction model using an RF model. During the training run, the model was adjusted for 200 iterations, and we used 10-fold cross-validation to evaluate the accuracy of the model training results. After the model training, we input 15 modeling factor layers and simulated SOCD on each raster to obtain the national SOCD layers of 1982, 1990, 2000, 2010, and 2020. At the same time, we calculated the interquartile (the difference between the 75th and 25th percentiles) of 200 iterations of simulated SOCD for each raster, which was used to represent the uncertainty of the simulated SOCD (Ding et al., 2016) (**Supplementary Figure 1**). The Chinese grassland SOCD layers were extracted by mask using the Chinese grassland vector layers. Following Liang et al. (2019), we examined the spatial simulation performance and validated the spatial accuracy of the grassland SOCD generated from the RF model using 100 measured samples from the original data and their corresponding values from the 2010 grassland SOCD layer. As there were few measured samples for a single year to be obtained, the temporal accuracy of the grassland SOCD generated from the RF model was validated as follows: we selected the measured samples between 2000 and 2005 (*n* = 100) and treated these soil samples as the measured samples in 2000. Then, we extracted the values of grassland SOCD layer in 2000 and their corresponding measured samples to test the performance of the model following Li H et al. (2022).

The observed data were used as the initial data to determine the contribution of each factor to grassland SOCD using the RF model. The factors with cumulative contributions larger than 70% were the dominant factors driving grassland SOCD change. For the dominant dynamic factors, using the grassland vector layers, we mask-extracted these factor layers to obtain the factor layers for the grassland section and used the zonal statistics (mean value calculation) to calculate the mean values of factors for the four regions (northwestern, northern, southern, and Qinghai–Tibetan regions). Then, we examined the trends of these factors over the past 39 years in four regions using the MK test. The mask extraction and zonal statistics were carried out on ArcGIS 10.2. The RF model and MK trend test were run on the R platform (R version 4.1.2, R Core Team, 2022), using the “randomForest” (Liaw and Wiener, 2002) and the “trend” (Pohlert, 2020) package, respectively.

## 3 Results

### 3.1 Random forest regression and factors’ contribution ranking

**Figure 3:**
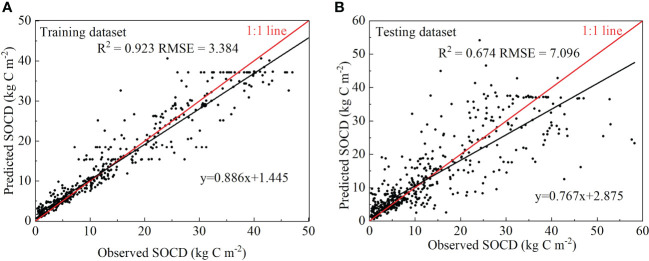
Accuracy evaluation of the model training dataset **(A)** and 10-fold cross-validation **(B)** on the comparisons between the model’s observed and predicted values for SOCD (0–100 cm) of grasslands. SOCD, soil organic carbon density; *R* ^2^, the coefficient of determination; RMSE, root of mean square error.

**Figure 4:**
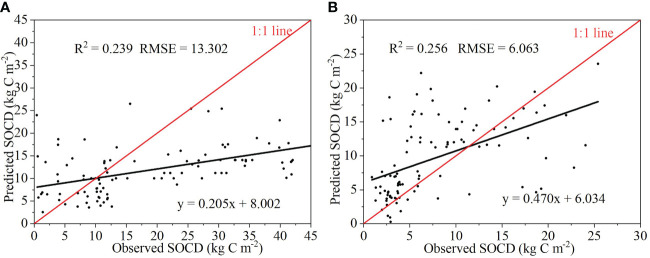
**(A)** is the validation result of 100 measured samples in 2010 with the corresponding simulated values of the mean grassland SOCD in 2010; **(B)** is the verification results of 100 measured samples (from 2000 to 2005) and their corresponding simulated values of the mean grassland SOCD in 2000. SOCD, soil organic carbon density; *R* ^2^, the coefficient of determination; RMSE, root mean square error.

The accuracy of the RF model training dataset and the results of 10-fold cross-validation are shown in **Figure 3**. The *R* ^2^ of the training dataset was 0.923 and the RMSE was 3.384 kg C m^−2^ (**Figure 3A**). The results of the 10-fold cross-validation showed that the *R* ^2^ of the model was 0.674 and the RMSE was 7.096 kg C m^−2^ (**Figure 3B**). These results indicated a good correlation between observed SOCD and predicted SOCD. The *R* ^2^ and RMSE of the spatial accuracy of the grassland SOCD prediction are shown in **Figure 4A**, where the *R* ^2^ and RMSE were 0.239 and 13.302 kg C m^−2^, respectively. The temporal accuracy of the grassland SOCD prediction is presented in **Figure 4B**, with an *R* ^2^ of 0.256 and an RMSE of 6.063 kg C m^−2^. According to the contribution ranking of the RF model, we considered the top 4 factors (cumulative contribution > 70%) as the dominant factors driving changes in grassland SOCD, and they were MAT, NDVI, elevation, and MAWS (**Table 2**).

**Table 2:** Ranking of the contribution of modeling factors based on the random forest model.

	SOCD
	Contribution (%)
MAT	44.00
NDVI	13.45
Elevation	10.62
MAWS	5.16
LLP	4.43
Clay soil	4.30
aspect	2.95
Sand soil	2.91
ET	2.88
NPP	2.34
SH	2.25
RH	1.61
MAP	1.43
Silt soil	1.28
SP	0.39

SOCD, soil organic carbon density; MAT, mean annual temperature; NDVI, normalized difference vegetation index; MAWS, mean annual wind speed; LLP, large livestock population; NPP, net primary production; ET, evapotranspiration; SH, sunshine hours; MAP, mean annual precipitation; RH, relative humidity; SP, sheep population.

### 3.2 Spatial and temporal characteristics of SOCD (0–100 cm) in grasslands from 1982 to 2020

**Figure 5:**
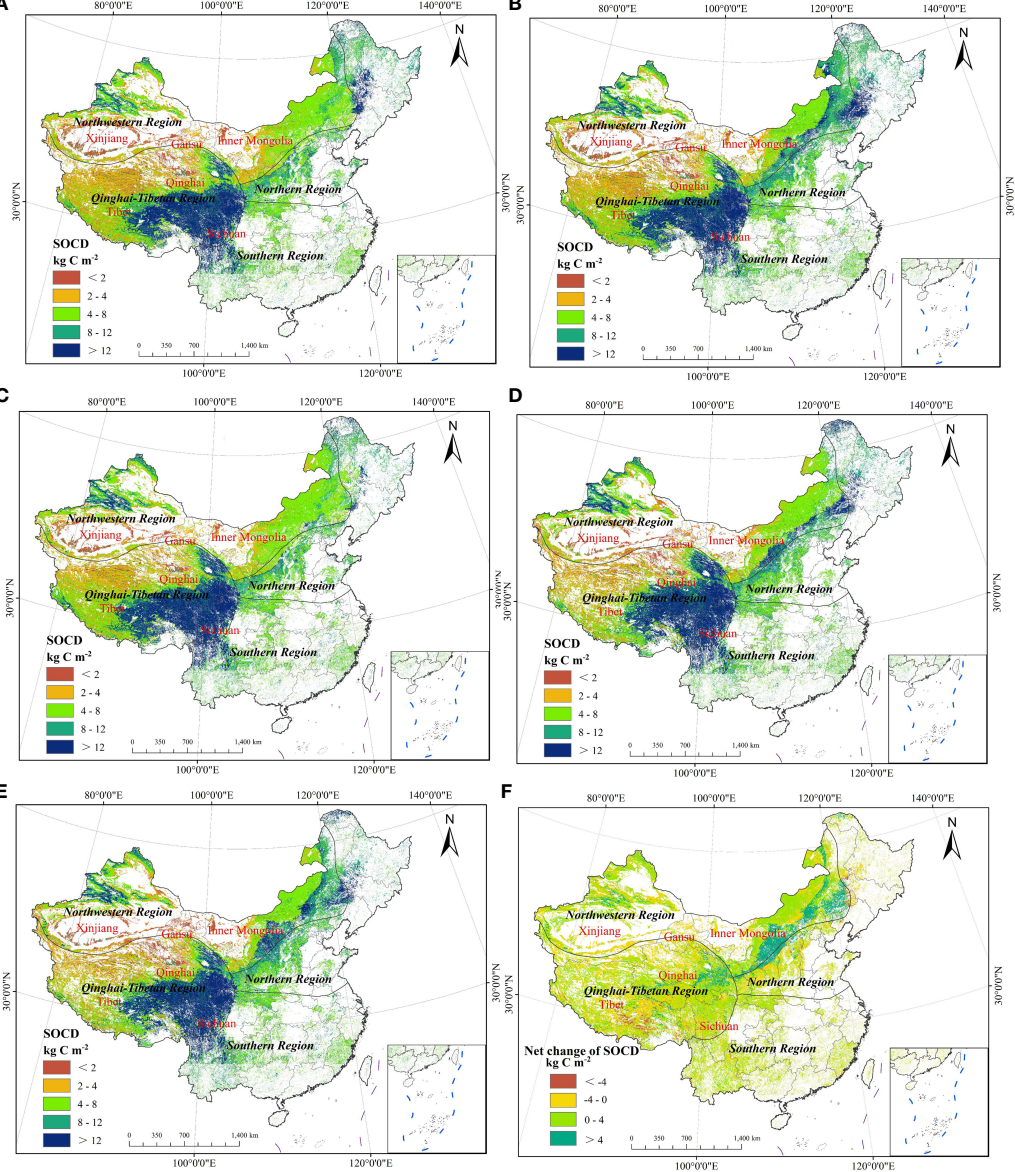
Spatial distribution of Chinese grassland average SOCDs in 1982 **(A)**, 1990 **(B)**, 2000 **(C)**, 2010 **(D)**, and 2020 **(E)**. SOCD was divided into five classes (SOCD<2 kg C m^−2^, 2–4 kg C m^−2^, 4–8 kg C m^−2^, 8–12 kg C m^−2^, and >12 kg C m^−2^). **(F)** is the net change of SOCD from 1982–2020, divided into four classes (<−4 kg C m^−2^, −4–0 kg C m^−2^, 0–4 kg C m^−2^, and >4 kg C m^−2^). SOCD, soil organic carbon density. The spatial distribution maps of Chinese grassland mean SOCD and the 25th and 75th percentile SOCDs in Chinese grasslands for each year are shown in **Supplementary Figure 1**.

At the national level, grassland SOCD simulated by the RF model was low in the west and north and high in the east and south (**Figure 5**). The high SOCD values were distributed in the eastern Qinghai–Tibetan region and near the Tianshan Mountains of the Xinjiang Uygur Autonomous Region, while the low SOCD values were mainly located in the western Qinghai–Tibetan region and central northwestern region. In the southern and northern regions, the values of grassland SOCD generally ranged from 4 to 12 kg C m^−2^. In the northwestern region, the SOCD values were relatively high in the eastern Inner Mongolia and northern Xinjiang (greater than 4 kg C m^−2^), and relatively low in the rest of this region (less than 4 kg C m^−2^). The spatial heterogeneity of grassland SOCD in the Qinghai–Tibetan region was large, showing a gradual increase from northwest to southeast. In this region, SOCD values were higher than 12 kg C m^−2^ in southern Tibet, southern Qinghai, and western Sichuan, and the values were lower than 12 kg C m^−2^ in the rest of the region. During the period 1982–2020, the largest net increase of SOCD occurred in eastern Inner Mongolia, with an increase over 4 kg C m^−2^. Conversely, the SOCD decreased the most in southern Tibet where the reduction was greater than 4 kg C m^−2^.

During the study period, the mean grassland SOCD increased in each period with respect to 1982 across China, with a net increase of 0.734 kg C m^−2^ from 1982 to 2020 (**Table 3**). However, variation in the mean grassland SOCD among regions was different over time. In the northern region, the mean grassland SOCD first decreased and then increased, and it was 9.471 kg C m^−2^ in 1982 and 9.299 kg C m^−2^ in 2020, decreasing by 0.172 kg C m^−2^. The mean grassland SOCD in the northwestern, Qinghai–Tibetan, and southern regions showed a net increase of 1.440 kg C m^−2^, 0.915 kg C m^−2^, and 0.411 kg C m^−2^, respectively. In the three regions, the grassland SOCD of each period showed varying degrees of increase compared to 1982.

**Table 3:** Estimated mean grassland SOCD (with interquartile range) (0–100 cm) (kg C m^-2^) for the four major geographic divisions in 1982, 1990, 2000, 2010, and 2020.

	1982	1990	2000	2010	2020
Southern region	8.892 (6.125–11.234)	9.056 (6.310–11.363)	9.086 (6.340–11.392)	9.213 (6.457–11.515)	9.303 (6.553–11.613)
Northern region	9.471 (6.949–11.647)	9.403 (7.029–11.407)	8.804 (6.524–10.751)	9.496 (7.118–11.516)	9.299 (7.021–11.216)
Northwestern region	5.504 (4.139–6.644)	6.924 (5.085–8.451)	5.907 (4.510–7.087)	6.395 (4.783–7.733)	6.943 (5.160–8.417)
Qinghai–Tibetan region	8.181 (5.506–10.445)	8.200 (5.579–10.436)	8.855 (6.092–11.212)	9.656 (6.683–12.180)	9.097 (6.277–11.483)
China	7.791 (5.529–9.710)	8.257 (5.898–10.244)	7.993 (5.753–9.891)	8.517 (6.136–10.521)	8.525 (6.152–10.516)

### 3.3 Changes in grassland area and SOCS (0–100 cm) from 1982 to 2020

Chinese grassland area showed a decreasing trend between 1982 and 2020, with a decrease of 3.985 × 10^5^ km² (**Table 4**). The most evident decrease in grassland area was in the Qinghai–Tibetan region, with a decrease of 2.494 × 10^5^ km². The least decrease in the area was observed in the southern region, with a reduction of 1.318 × 10^4^ km². Over the whole study period, an area of 8.549 × 10^5^ km² of grassland was mainly converted to unused land (58.14%), woodland (20.22%), and cropland (14.82%) (**Table 5**). In addition, there was also a small proportion of grassland converted to water body and built-up land.

**Table 4:** Chinese grassland area from 1982 to 2020 (km^2^).

	1982	1990	2000	2010	2020
Southern region	2.851×10^5^	2.867×10^5^	2.824×10^5^	2.714×10^5^	2.720×10^5^
Northern region	2.774×10^5^	2.688×10^5^	2.551×10^5^	2.329×10^5^	2.160×10^5^
Northwestern region	1.020×10^6^	1.019×10^6^	1.004×10^6^	9.468×10^5^	9.454×10^5^
Qinghai–Tibetan region	1.470×10^6^	1.469×10^6^	1.468×10^6^	1.221×10^6^	1.220×10^6^
China	3.052×10^6^	3.044×10^6^	3.009×10^6^	2.672×10^6^	2.654×10^6^

**Table 5:** Transformed grassland area from 1982 to 2020 (km^2^).

	Transformed area (km^2^)	Percentage (%)
	1982–2020	
Grassland–Built-up Land	1.521×10^4^	1.78
Grassland–Farmland	1.267×10^5^	14.82
Grassland–Woodland	1.728×10^5^	20.22
Grassland–Water Body	4.312×10^4^	5.04
Grassland–Unused Land	4.970×10^5^	58.14
Total changed area	8.549×10^5^	100

The total SOCS of Chinese grasslands showed a change featuring first an increase and then a decrease (**Table 6**). The increase of grassland SOCS was obvious during 1982–1990 with an increase of 1.352 Pg, then gradually decreased by 2.509 Pg during 1990–2020. During the study period, the net decrease of 1.158 Pg in grassland SOCS was mainly caused by the reduction from 1990 to 2020. In four regions, from 1982 to 2020, grassland SOCS of the northwestern region showed an overall upward trend, with a net increase (0.950 Pg), and it showed a net decrease in the southern (0.005 Pg), northern (0.618 Pg), and Qinghai–Tibetan regions (0.923 Pg), respectively. Among these three regions, grassland SOCS in the southern and Qinghai–Tibetan regions increased slightly during 1982–2000 and then decreased until 2020, while the northern region experienced a gradual decrease from 1982 to 2020.

**Table 6:** Soil organic carbon storage (with interquartile range) (Pg) in Chinese grasslands (0–100 cm) from 1982 to 2020.

	1982	1990	2000	2010	2020
Southern region	2.536 (1.746–3.203)	2.596 (1.809–3.258)	2.565 (1.790–3.217)	2.500 (1.752–3.125)	2.530 (1.782–3.158)
Northern region	2.627 (1.927–3.230)	2.527 (1.889–3.066)	2.246 (1.664–2.743)	2.211 (1.658–2.682)	2.009 (1.517–2.423)
Northwestern region	5.614 (4.222–6.778)	7.056 (5.182–8.612)	5.928 (4.525–7.112)	6.054 (4.529–7.321)	6.564 (4.879–7.957)
Qinghai–Tibetan region	12.023 (8.092–15.350)	12.049 (8.197–15.335)	13.000 (8.943–16.460)	11.788 (8.158–14.869)	11.100 (7.660–14.013)
China	23.780 (16.875–29.637)	25.132 (17.952–31.181)	24.051 (17.311–29.765)	22.757 (16.395–28.110)	22.623 (16.326–27.907)

## 4 Discussion

### 4.1 Reasons for differences in grassland’s SOCD among different regions

Chinese grassland SOCD was high in the east and south, and low in the west and north (**Figure 5**), a similar observation reported by Liu et al. (2022). Generally, SOCD is influenced by biological productivity and organic matter mineralization, as well as controlled by hydrothermal conditions (Duchaufour, 1983; NSSO, 1998). In the southern region of the study area, the climate is more humid due to monsoonal circulation (Zhang, 1991; Wu and Peng, 2003). On the contrary, in the northwestern region, the climate condition is arid due to the far distance from the ocean and the blockage of the Qinghai–Tibetan plateau (Wu and Peng, 2003). In the southern and northern regions with a warm climate and abundant precipitation, improved vegetation growth and high primary productivity promote the accumulation of SOC and contribute to higher grassland SOCD (Ontl and Schulte, 2012). In northwestern China, the western part is mainly covered by desert, while the eastern part is mostly covered by grasslands. The dry climatic conditions and scarce precipitation are not conducive to vegetation growth and high NPP, which might contribute to relatively low SOCD in the western part (Li et al., 2004; Ontl and Schulte, 2012; Zhang et al., 2016). The Qinghai–Tibetan region has unique climatic conditions, and the elevation of this region is high in the west and low in the east (Zhang Y et al., 2022; Liu et al., 2019). The cold and relatively humid conditions promoted the accumulation of soil organic matter in the southeastern of the Qinghai–Tibetan region, resulting in higher SOCD (Piao et al., 2011; Zhong et al., 2012).

### 4.2 Dominant factors determining SOCD

The mean SOCD of Chinese grasslands was 7.791 kg C m^−2^ in 1982 and 8.525 kg C m^−2^ in 2020, showing a net increase of 0.734 kg C m^−2^ (**Table 3**). This was consistent with our hypothesis that grassland SOCD showed a net increasing trend. The results from the RF model suggested that MAT, NDVI, elevation, and MAWS were the main factors driving the variation in grassland SOCD across China, with MAT being the most important factor, explaining 44.00% of the total variation, followed by NDVI, elevation, and MAWS, explaining 13.45%, 10.62%, and 5.16%, respectively (**Table 2**).

#### 4.2.1 Effects of MAT on SOCD

**Figure 6:**
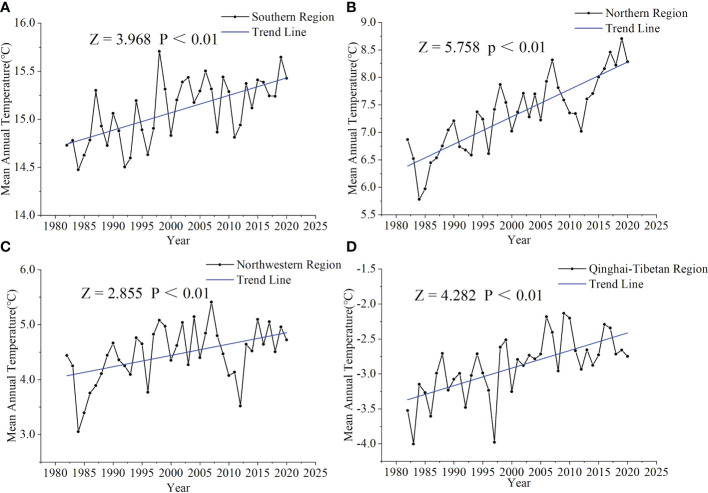
Trends in mean annual temperature from 1982 to 2020 in the southern **(A)**, northern **(B)**, northwestern **(C)**, and Qinghai–Tibetan **(D)** regions. *Z* > 0 or *Z*< 0 indicates an increasing or decreasing trend, respectively. *p*< 0.01 indicates a significant increasing or decreasing trend.

In the northern region of China, MAT showed an increasing trend, resulting in a net decrease in SOCD in the region (**Table 3**; **Figure 6B**). This is due to the fact that (i) the fine and coarse root mass quantity and quality reduced with increased temperature, and the decline in root mass was an important cause of the loss in soil organic matter (Rasse et al., 2005; Ofiti et al., 2021), and (ii) the temperature rise would accelerate the decomposition of soil organic matter and thus reduce SOCD (Duan et al., 2013; Lv et al., 2020; Li B et al., 2022). However, in the southern, northwestern, and Qinghai–Tibetan regions, the climate warming seemed to increase SOCD (**Table 3**; **Figures 6A, C, D**). This was probably because, on the one hand, although an increased temperature accelerated the release of soil carbon into the atmosphere (Davidson and Janssens, 2006; Leblans et al., 2017), on the other hand, it contributed to a longer growing season (Leblans et al., 2017) and stimulated plant productivity and thus increasing litter input into the soil (Piao et al., 2006). When the rate of soil carbon input exceeds the rate of soil carbon decomposition, SOCD will gradually increase (Chen et al., 2016; Nie et al., 2019).

#### 4.2.2 Effect of vegetation cover on SOCD

**Figure 7:**
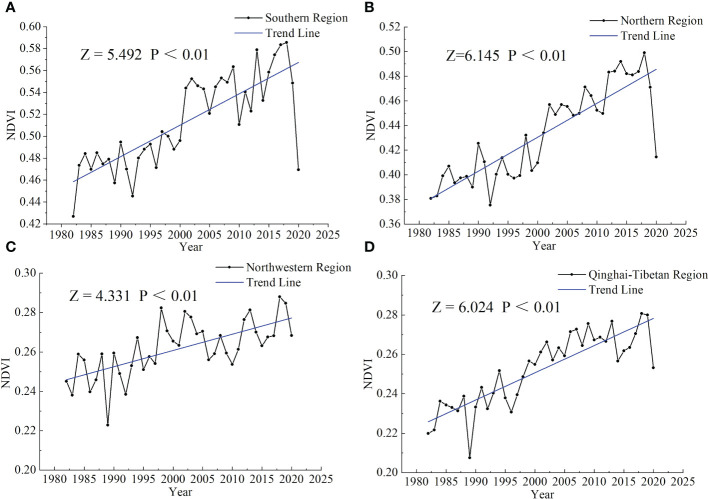
Trends in NDVI from 1982 to 2020 in the southern **(A)**, northern **(B)**, northwestern **(C)**, and Qinghai–Tibetan **(D)** regions. *Z* > 0 or *Z*< 0 indicates an increasing or decreasing trend, respectively. *p*< 0.01 indicates a significant increasing or decreasing trend. NDVI, normalized difference vegetation index.

NDVI is an important indicator of vegetation cover. Changes in NDVI represent the changes in vegetation cover, which plays an important role in SOC accumulation (Zhang C et al., 2021; Wang et al., 2018). In the southern, northwestern, and Qinghai–Tibetan regions, NDVI gradually increased over the years (**Figures 7A, C, D**), resulting in a net increase in SOCD in these regions (**Table 3**). With a gradual increase in NDVI, the vegetation cover also increased (Tian et al., 2022). Studies have proven that an increase in vegetation cover could facilitate soil carbon accumulation and thus increase SOCD (Smith, 2008; Don et al., 2011; Gong et al., 2017). This can be ascribed to (i) an increase in NPP of plant roots (Smith, 2008; Gong et al., 2017), (ii) a reduction in the loss of SOC by effectively blocking wind erosion (Don et al., 2011), and (iii) the accumulation of litter on the soil surface (Li et al., 2020). However, in the northern region, NDVI gradually increased, while SOCD showed a net decrease (**Figure 7B**; **Table 3**). Generally, increase in vegetation is followed by an increase in the litter (Tian et al., 2022). As the organic matter from the litter input to the soil increases, it may result in a “priming effect”. That is to say, soil microbes are supposed to be incentivized by readily decomposable organic matter, and CO_2_ emission from soil increases disproportionately, resulting in the loss of SOC (Kuzyakov et al., 2000; Heimann and Reichstein, 2008; Sayer et al., 2011). In summary, the relationship between NDVI and SOCD is complicated and needs further study.

#### 4.2.3 Effect of MAWS on SOCD

**Figure 8:**
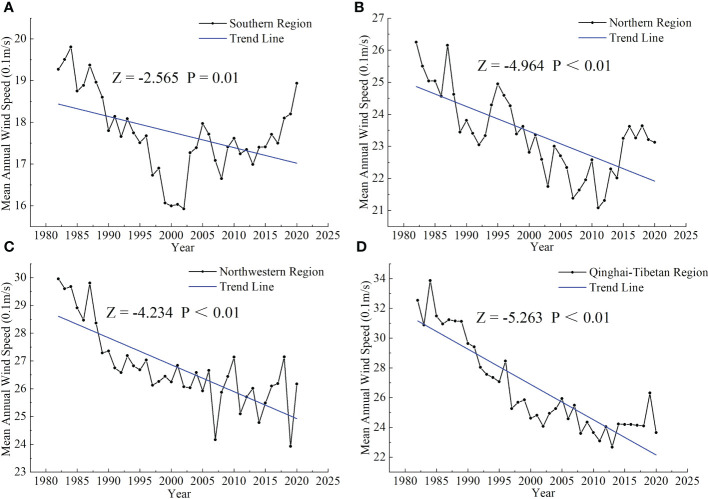
Trends in mean annual wind speed from 1982 to 2020 in the southern **(A)**, northern **(B)**, northwestern **(C)**, and Qinghai–Tibetan **(D)** regions. *Z* > 0 or *Z*< 0 indicates an increasing or decreasing trend. *p*< 0.01 indicates a significant increasing or decreasing trend, respectively.

Annual variation in wind speed directly affects wind erosion intensity (Wu et al., 2021). Wind erosion is a natural process that affects ecosystems and is more pronounced in arid and semi-arid regions (Shao et al., 2011; Lei et al., 2019). It can cause loss of organic carbon by transferring soil organic matter from the soil surface to the atmosphere (e.g., dust) (Zhang W et al., 2022; Lal, 2003; Borrelli et al., 2017). In the northwestern region, a significant decreasing trend in MAWS led to a reduction in wind erosion intensity, promoting the accumulation of SOCD in the region (**Table 3**; **Figure 8C**; Wu et al., 2021). In addition, studies showed that adequate wind speed will promote intercellular CO_2_ exchange, resulting in an increase in intercellular CO_2_ concentration and accelerating the rate of net phosphate synthesis in plants, thereby increasing plant NPP (Xu et al., 2017) and the accumulation of organic matter in the soil. This might be the reason that SOCD increased in the southern region (**Table 3**; **Figure 8A**). In the Qinghai–Tibetan region, SOCD showed a net increase (**Table 3**). This can be attributed to two aspects: (i) wind erosion is positively correlated with wind speed, and reduced MAWS weakened the power of wind erosion (**Figure 8D**; Wu, 2003; Zhang C et al., 2018); (ii) a range of ecological projects (such as Returning Grazing Land to Grassland Program) that have been implemented on the Qinghai–Tibetan Plateau helped improve vegetation cover and soil fertility, thereby mitigating wind erosion (Li et al., 2016b; Chen et al., 2014). The northern region is in a semi-humid climate where 70% of the annual precipitation falls in summer, resulting in frequent flooding and reduced vegetation growth (Zhang Y et al., 2021). As a result, although there was a significant downward trend of MAWS in the northern region, SOCD decreased significantly over the past decades (**Table 3**; **Figure 8B**).

#### 4.2.4 Effects of elevation on SOCD

In general, topographic factors do not directly influence SOC accumulation, but they can influence vegetation types and soil hydrothermal conditions by regulating climate, which, in turn, affects vegetation productivity and soil organic matter decomposition (Cao et al., 2020; Tian et al., 2022). As elevation increases, the decrease in temperature leads to a reduction in the decomposition rate of soil organic matter, which facilitates SOC accumulation and thus increases SOCD (Chen et al., 2013). However, it was also found that in warmer areas at low latitudes, SOC increased with elevation, while in colder areas at high latitudes, SOC decreased with elevation (Yin et al., 2022). This implies that the effect of elevation on SOCD is complex and requires further study.

### 4.3 Grassland area change effects on soil organic carbon storage

**Figure 9:**
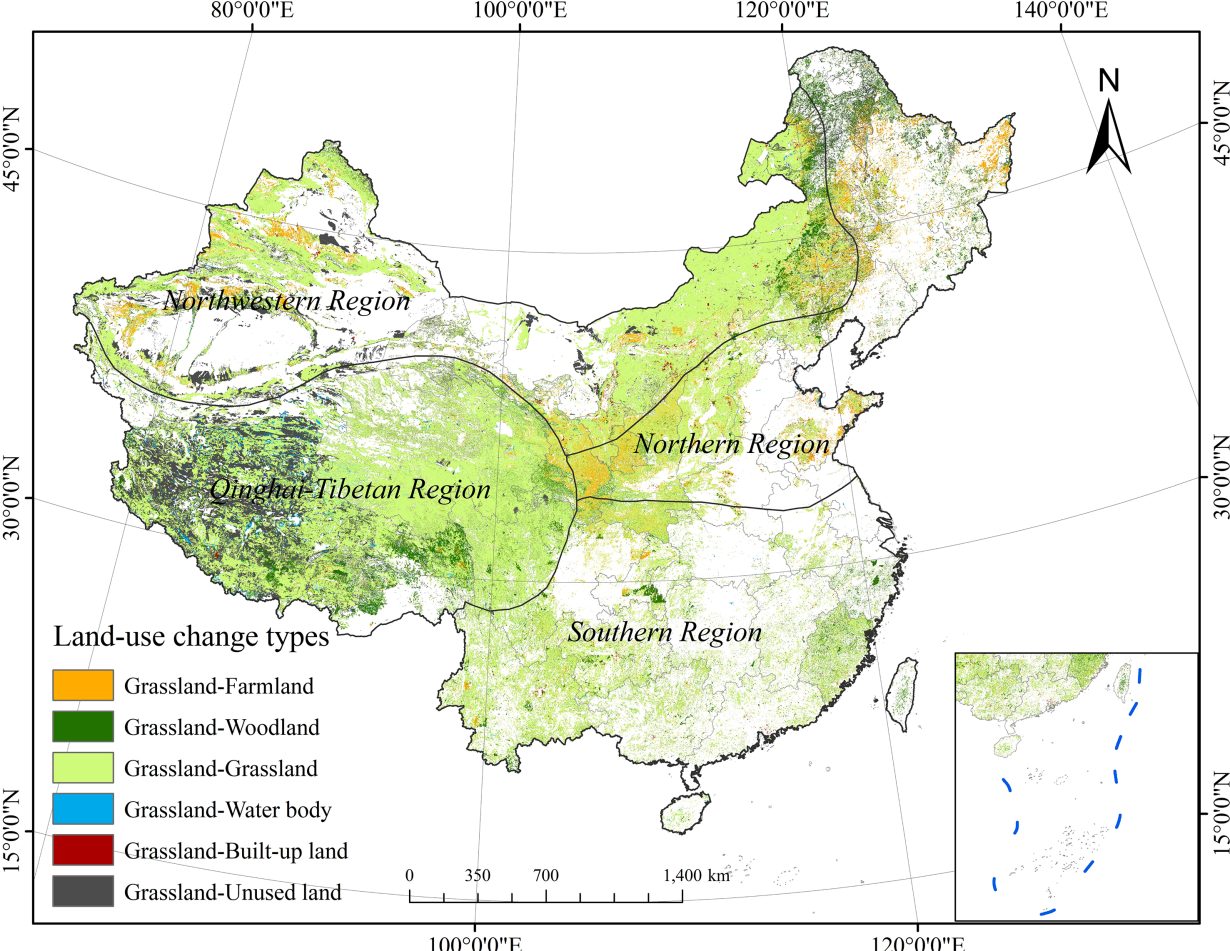
The spatial distribution of grassland transformed from 1982 to 2020 (km^2^).

Grassland SOCD and grassland area are important factors influencing changes in grassland SOCS. A reduction in grassland area would lead to the loss of grassland SOCS and make grasslands a source of greenhouse gas emissions. Between 1982 and 2020, the Chinese grassland area declined severely, with a total reduction of 3.985 × 10^5^ km^2^, resulting in a 1.158 Pg reduction in grassland SOCS (**Tables 4** and **6**). This was contrary to our hypothesis that SOCS of Chinese grasslands increased between 1982 and 2020. The transfer of grasslands to unused lands was the main reason for the reduction in grassland area, accounting for 58.14% of the total changed area (**Table 5**; **Figure 9**). Various factors, such as population growth, overgrazing, emphasis on livestock over grass, frequent droughts, insects, and other natural disasters, accelerate the process of grassland degradation and desertification (Bardgett et al., 2021; Li et al., 2021). These led to the conversion of some grasslands to unused lands (gobi, sandy land, swampland, gravel and rock, bare land, and other unused lands) and resulted in the loss of SOCS. Furthermore, the cultivation of grassland, the construction of reservoirs, and the development of urban areas led to the conversion of some grasslands to croplands, water bodies, and built-up lands (Bardgett et al., 2021; Chang et al., 2022; Li et al., 2016a). Different land types have different SOCDs, and it is generally accepted that woodlands have the highest SOCD, followed by grasslands, croplands, and built-up lands (Li Y et al., 2022; Lin et al., 2021; Zhu et al., 2021). Therefore, the conversion of grasslands to woodlands with high SOCD usually promoted the accumulation of SOCS. In contrast, the conversion of grasslands to land types with low SOCD (e.g., croplands, water bodies, and built-up lands), resulted in a loss of SOCS, leading to significant CO_2_ emission to the atmosphere and accelerating global warming.

### 4.4 Countermeasures and suggestions

As mentioned above, the SOCD of grasslands in China gradually increased during the study period. This implies that more carbon can be sequestrated into the soil of grasslands. However, due to the reduction in grassland area caused by degradation and conversion, grassland SOCS decreased across China in general. Therefore, taking certain measures to mitigate the decline in grassland areas appears to be crucial in increasing grassland SOCS.

To do this, China has implemented several ecological restoration projects since the 1980s, including the Grain to Green Program introduced in 1999 and the Returning Grazing Land to Grassland Program introduced in 2003 (Li et al., 2016a; Zhou W et al., 2017). These programs were aimed to convert agricultural lands into forests and grasslands (Ferraro and Kiss, 2002) and to relieve grazing pressure on degraded grasslands through grazing bans, fallow grazing for grass, fencing, and rotational grazing (Tong et al., 2004). It was shown that the carbon sequestration amount due to these programs’ implementation since 2001 and 2010 was 0.200 Pg and 0.118 Pg, respectively (Lu et al., 2018). Various grassland protection programs, such as banning grassland reclamation and private conversion and destruction of grasslands, were also enforced (Xing, 2016). These measures not only were effective in alleviating the degradation of grasslands and the reduction of SOCS due to grassland area decrease (Li et al., 2016a; Zhou W et al., 2017), but also enhanced the grassland’s ability against wind erosion through increasing vegetation cover (Shao et al., 2016).

Besides this, as temperature and altitude influenced grassland SOCD change, additional work should be done in the future. For example, we should pay more attention to temperature or altitudes that caused a decrease in SOCD. We should try to restrict change from carbon sources to pools in such areas by implementing proper ecological engineering or adopting measures such as water and soil conservation and regulating grassland use practices. However, rarely has any study looked at the improvement of grassland SOCD in specific temperatures and altitudes.

### 4.5 Uncertainty and limitation of the study

The RF model has been used extensively for modeling. For example, Li H et al. (2022) and Sreenivas et al. (2014) used it to quantify the dynamics of SOC in China and SOCD in southern India, respectively. Their findings demonstrated the feasibility of the model in predicting SOC dynamics. In this study, the results also indicated that the RF model has good spatiotemporal predictive power (**Figure 4**). However, there are still some uncertainties in the model.

First, there may be more factors that contribute to SOCD changes but were not included in our RF model. These factors include litter quality, nitrogen deposition, and CO_2_ enrichment (Zhang W et al., 2022; Sumiyoshi et al., 2017). Future studies should focus on including more factors in the model and intensive sampling in sparsely sampled areas to reduce the uncertainty of model predictions (Liang et al., 2019). Second, the data sources are not consistent. For example, the data sources of some modeling factors used to predict grassland SOCD in 2020 were not consistent with the previous data sources, which may affect the model prediction results (Zhang et al., 2017). Third, we only have the observed soil samples data in 2010 to build a model, which may cause some uncertainty in the results over a long time series of simulations. Fourth, while the datasets containing 552 samples across the study area are large enough to increase the performance of the model and extrapolability of the results, assessment of the spatial coverage of samples was not carried out and is beyond the scope of the study objective. Therefore, in future studies, assessment of spatial coverage of sample locations to capture the variability of environmental conditions within the four regions (e.g., the southern, northern, northwestern, and Qinghai–Tibetan regions) should be considered to increase the extrapolability of the results.

## 5 Conclusions

From 1982 to 2020, the spatial distribution of SOCD (0–100 cm) in Chinese grasslands showed little change, with low SOCD in the west and north and high SOCD in the east and south. The absolute quantity of Chinese grassland SOCD showed a net increase during the study period. The grassland SOCD showed a net decreasing trend in the northern region, and a net increasing trend in the southern, northwestern, and Qinghai–Tibetan regions. The MAT was the most important factor driving the variation in SOCD (its contribution rate accounting for 44.00%) of Chinese grasslands, followed by NDVI, elevation, and MAWS.

Due to the reduction in grassland area over the last 39 years, the total organic carbon storage of Chinese grassland soils was 22.623 Pg in 2020, a net decrease of 1.158 Pg compared to that in 1982. Land-use change is the reason for the decline in grassland area, and the conversion of grasslands to other land types such as croplands, built-up lands, and unused lands usually leads to large amounts of CO_2_ emission into the atmosphere, exacerbating global warming. Therefore, there is an urgent need to protect grasslands, and to prevent grasslands from degradation and insist on the implementation of ecological restoration programs. In addition, the conversion of grasslands to other land types should be avoided as far as possible to reduce the release of soil carbon.

## Data availability statement

The original contributions presented in the study are included in the article/**Supplementary Material**. Further inquiries can be directed to the corresponding author.

## Author contributions

JJC, JC, AB, HS, SH, HW, and XD contributed to manuscript writing. JJC, JC, and AB revised the manuscript. All authors contributed to the article and approved the submitted version.
