# Supplementary material for: Quantifying changes in soil organic carbon density from 1982 to 2020 in Chinese grasslands using a random forest model
Source: Front Plant Sci. 2023 May 8;14:1076902. doi: 10.3389/fpls.2023.1076902 (PMC10316965; doi:10.3389/fpls.2023.1076902)
Supplement: Supplementary file 1 [file DataSheet_1.docx]

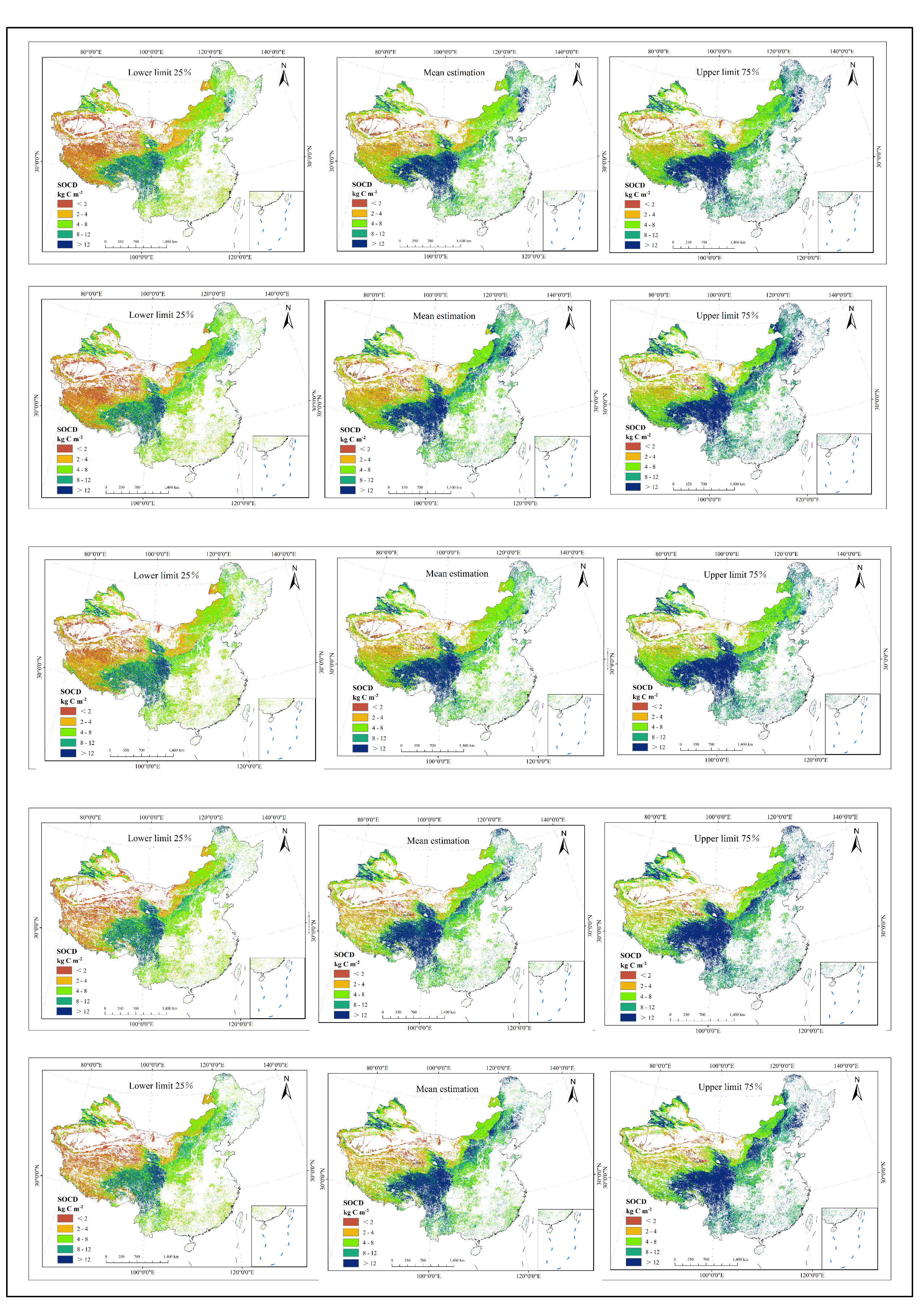


**Supplementary Figure 1.** The spatial distribution maps of Chinese grassland mean SOCD and the 25th and 75th percentile SOCD in Chinese grasslands in 1982, 1990, 2000, 2010, 2020. SOCD, soil organic carbon density.
